# Direct Observation of Ascorbyl Free Radicals in Whole Blood of Goats During Cardiac Surgery With Cardiopulmonary Bypass

**DOI:** 10.7759/cureus.95067

**Published:** 2025-10-21

**Authors:** Osamu Tokumaru, Shigekiyo Matsumoto, Takayuki Mizoguchi, Kazue Ogata, Keitaro Okamoto, Takayuki Kawashima, Hirofumi Anai, Shinji Miyamoto, Takaaki Kitano

**Affiliations:** 1 Faculty of Welfare and Health Sciences, Oita University, Oita, JPN; 2 Anesthesiology, Oita University Faculty of Medicine, Oita, JPN; 3 Clinical Engineering, Oita University Hospital, Oita, JPN; 4 Cardiovascular Surgery, Oita University Faculty of Medicine, Oita, JPN

**Keywords:** ascorbate, cardiopulmonary bypass, electron spin resonance, free radical, open-heart surgery

## Abstract

Background: Ascorbate is located at the most downstream end of radical scavenging reactions and reacts with various free radicals to form ascorbyl free radical (AFR). In this study, we assessed AFR levels in whole blood samples obtained from perioperative goats under cardiac surgery with cardiopulmonary bypass (CPB) using electron spin resonance (ESR) spectrometry without adding any spin trap, which the stability of AFR made possible. The direct radical scavenging activity of the plasma was evaluated for hydroxyl radicals by the spin trapping method.

Materials and methods: This is an experimental animal study on goats (n = 19). Blood samples were collected at eight time points during the heart surgery using CPB. The whole blood sample was aspirated in a disposable ESR flat cell, and 95% confidence intervals (95% CIs) of the amount of AFR relative to those of the induction of anesthesia were quantified using ESR spectroscopy at each time point of the heart surgery. The scavenging activity of the plasma against hydroxyl radical and DPPH (2,2-diphenyl-1-picrylhydrazyl) was also evaluated using ESR spectroscopy with the spin trapping method; a dose-response curve for each free radical was drawn to estimate the half maximal inhibitory concentration (IC_50_) of plasma during heart surgery. The reciprocals of IC_50_ were used as the indicators of scavenging activities.

Results: The AFR level detected in goat blood significantly increased from the start of CPB (95% CI: 1.09-1.39 ) and remained significantly higher than the preoperative level during aortic clamping (1.15-1.73 at aortic cross-clamping, 1.17-1.74 one hour after clamping, and 1.16-1.52 two hours after clamping). The AFR returned to the preoperative levels after aortic declamping. The free radical scavenging activities of plasma sampled at aortic cross-clamping and from two hours after clamping to the end of surgery significantly increased against hydroxyl radicals (p < 0.05). Scavenging activity was not observed against DPPH.

Conclusion: Real-time assessment of oxidative stress was successfully conducted by ESR spectroscopy of whole blood without a spin trap. The biphasic increase in AFR during the open-heart surgery might reflect the production of free radicals under oxidative stress due to the cardiac surgery. The first phase might be due to inflammatory responses after CPB induction, and the second might reflect production of free radicals after reperfusion. We believe that measuring AFR levels in fresh whole blood would be a simple but informative indicator of oxidative stress during surgery.

## Introduction

Free radicals play a major role in the pathophysiology of various diseases, including ischemia/reperfusion injury, inflammation, and surgical stress. Anesthetic procedures, surgical invasion, and surgical intervention such as hemodialysis, extracorporeal membrane oxygenation, or cardiopulmonary bypass (CPB) during open-heart surgeries may trigger systemic inflammatory reactions and activate neutrophils to produce large amounts of free radicals, causing perioperative oxidative stress [1,2]. Anesthetic agents such as sevoflurane could modulate oxidative stress via preconditioning mechanisms. Excessive free radicals damage cellular components, such as nucleic acids, lipids, and proteins, and impair cellular functions; therefore, they are correlated with postoperative multi-organ failure and deteriorated prognosis. Recently, several studies have suggested that perioperative oxidative stress is correlated with complications after open-heart surgeries [1-3].

Direct detection of free radicals is difficult because of their high reactivity and short lifetimes. The spin-trapping method is used to detect free radicals using electron spin resonance (ESR) spectroscopy *in vitro* [4]. However, it is difficult to apply this method to observe free radicals produced *in vivo* [5].

Ascorbate is a water-soluble antioxidant present at the most downstream end of oxidative chain reactions. Ascorbate in extracellular fluid receives an unpaired electron from intracellularly generated free radicals via vitamin E embedded in the cell membrane, transforming itself to a stable ascorbyl free radical (AFR) [6,7]. Thus, AFR is considered the final product of intracellularly produced free radicals.

In the present study, we aimed to quantify AFRs in whole blood collected from perioperative goats. The main objectives of this study were (1) to demonstrate the direct detection of AFR in the blood of experimental animals undergoing open-heart surgery with CPB and (2) to evaluate the potency of AFR as a clinical indicator of perioperative oxidative stress during open-heart surgery.

## Materials and methods

Ethical approval

Animal care was conducted according to the “Guide for the Care and Use of Laboratory Animals,” published by the US National Institutes of Health (NIH publication no. 85-23, revised 1996) [8]. All animal experiments in this study were approved by the Animal Ethics Committee of Oita University (Protocol No.: 1622001).

Materials

CYPMPO (5-(2,2-dimethyl-1,3-propoxy cyclophosphoryl)-5-methyl-1-pyrroline N-oxide) was purchased from Mikuni Pharmaceutical Industrial Co., Ltd. (Osaka, Japan). DPPH (2,2-diphenyl-1-picrylhydrazyl) was purchased from Sigma-Aldrich (St. Louis, MO). Ethylenediaminetetraacetic acid (EDTA) was purchased from Dojindo (Kumamoto, Japan). Hydrogen peroxide was purchased from Wako Pure Chemical Industries, Ltd. (Osaka, Japan). All other reagents used in this study were of the highest commercially available quality.

Open-heart surgery in goats

**Figure 1:**
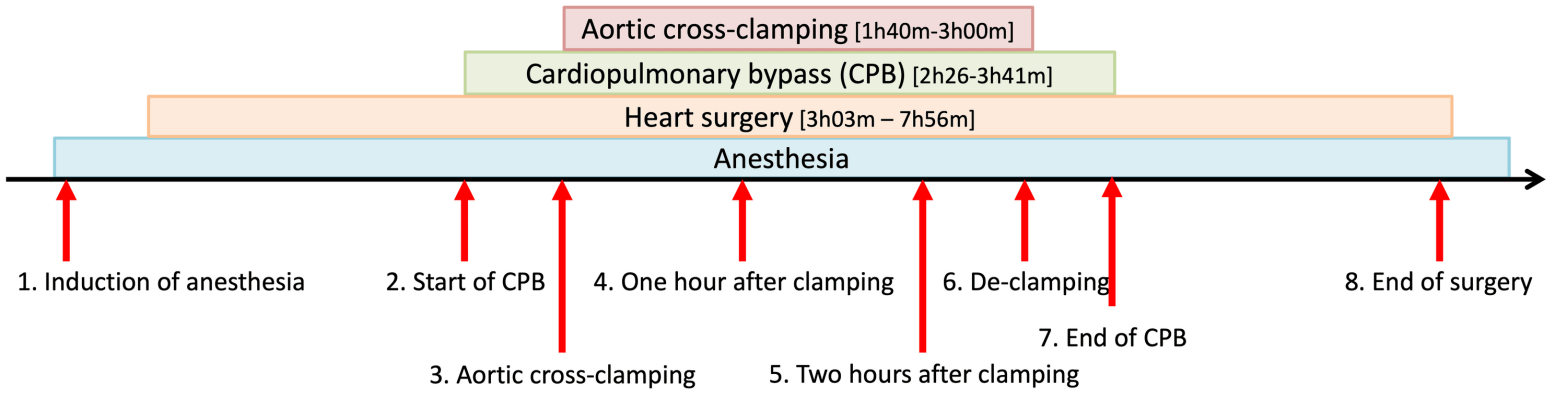
Experimental protocol. Blood was sampled at eight time points during cardiopulmonary bypass (CPB) surgeries (indicated by red arrows).

Goats can produce ascorbate *in vivo*. In this study, open-heart surgeries were performed on goats (n = 19) by cardiovascular surgeons (KO, TKa, HA, and SMi) through CPB in an experimental operating room for large animals similar to those used for human surgeries. General anesthesia was uniformly induced with ketamine hydrochloride (20 mg/kg intramuscularly administered) for sedation and analgesia, and maintained with sevoflurane (2-3%) across all animals. During CPB (performed by TM), the partial pressure of oxygen (P_O2_) was maintained between 250 and 350 Torr. The body temperature was maintained at 32°C to match the human clinical settings. Means (standard deviations) of operation time, duration of CPB, and aortic cross-clamp time were 335 (73) minutes, 180 (23) minutes, and 127 (19) minutes, respectively. Mannitol was administered at priming (300 mL) and de-clamping (100 mL). Blood samples were collected at eight time points during surgery (indicated by red arrows in Figure 1). All experimental animals recovered after surgery.

Detection of AFRs in whole blood

Blood was collected using a 2.5 mL syringe (Terumo, Tokyo, Japan) and transferred to a plastic sample tube containing an EDTA solution. The blood sample was then aspirated into a disposable ESR flat cell (Radical Research Inc., Tokyo, Japan) without adding the enhancer (dimethyl sulfoxide) [9]. The sample was inserted in the cavity of an X-band ESR spectrometer (JES-RE1X; JEOL, Tokyo, Japan) with the operation software WIN-RAD version 1.20b (Radical Research Inc.). The typical instrumental settings were as follows: temperature, 23°C; frequency, 9.45 GHz with 100-kHz modulation; modulation width, 0.1 mT; time constant, 0.1 s; center field, 335.5 mT; sweep width, 7.5 mT; sweep time, one minute; and accumulation, four times. The microwave power was set at 4 mW to ensure that the ESR signals were not saturated. The time from blood sampling to measurement was less than five minutes at most. Since the oxidation starts immediately after the sampling, the blood was sealed and transferred to the laboratory room as fast as possible to avoid possible oxidation due to exposure to air or a change in temperature.

Evaluation of free radical scavenging activity of perioperative plasma

In previous reports on CPB-related oxidative stress, many studies evaluated hydroxyl radicals. Therefore, we evaluated the scavenging activity of plasma against hydroxyl radicals in this study. An artificial free radical, DPPH, was also evaluated. The blood sample was centrifuged, and the plasma was stored at −80°C until the experiment. The direct free radical scavenging activity of perioperative goat plasma against hydroxyl and DPPH radicals was evaluated, following the methods described in previous studies [10]. Briefly, hydroxyl radicals were produced by ultraviolet (UV) irradiation with diluted hydrogen peroxide (100 mM H_2_O_2_) and trapped by CYPMPO. DPPH dissolved in ethanol (15 μM) was mixed with goat plasma. Free radicals were quantified by ESR spectrometry. ESR signals of manganese (II) oxide (MnO) were used as external references. The ratio of the height of the target signal of the free radical to that of MnO was calculated and standardized relative to the control ESR signal (without plasma addition). The reciprocals of half maximal inhibitory concentration (IC_50_) were used as the indicators of scavenging activities.

Statistical analysis

Statistical tests were performed using R version 4.3.0. (https://www.R-project.org/; R Foundation for Statistical Computing, Vienna, Austria). Values at each time point are presented as means and their 95% confidence intervals (95% CIs). No comparisons among time points were made. The level of significance was set at 0.05.

## Results

AFR levels detected in whole blood

**Figure 2:**
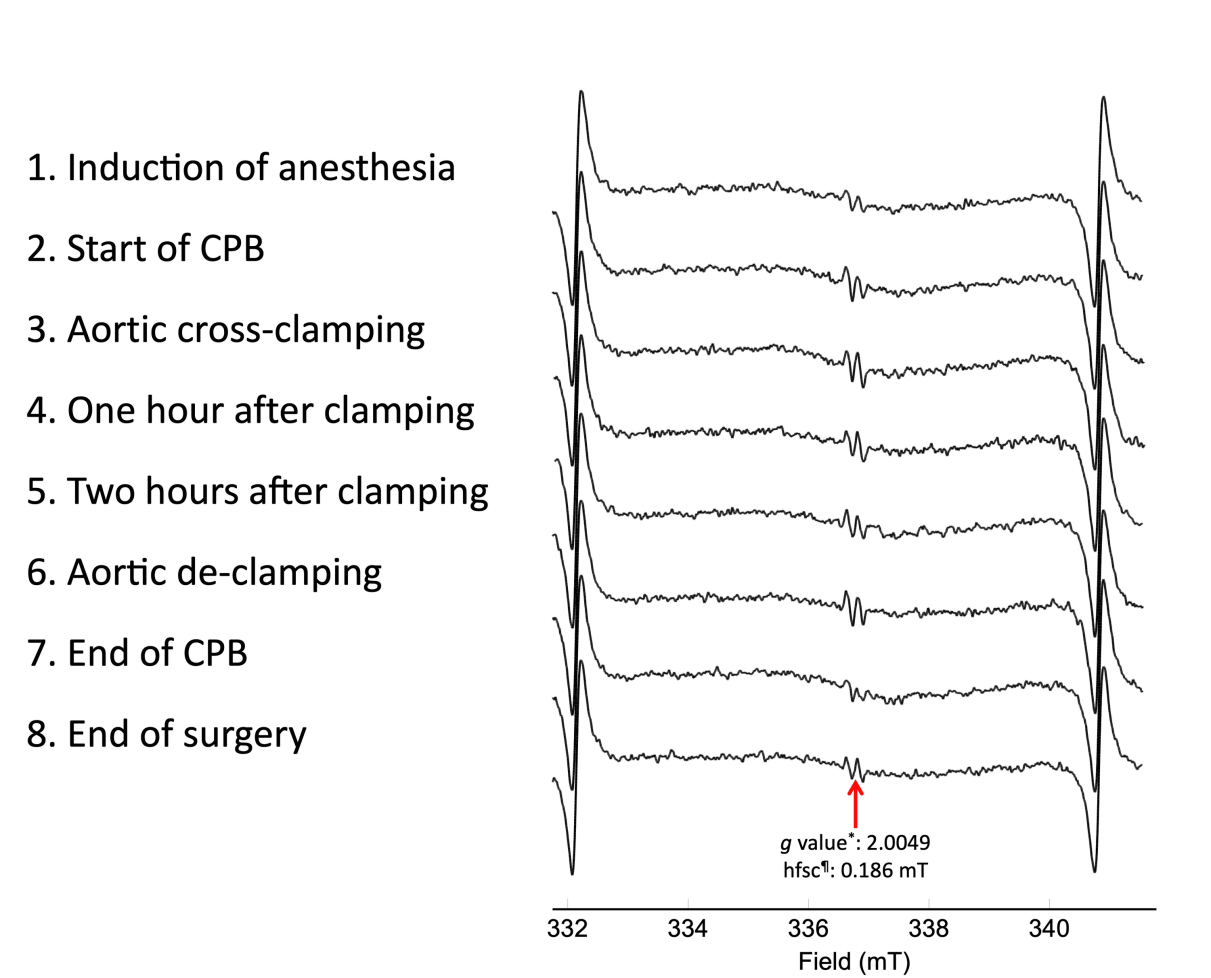
Representative electron spin resonance (ESR) spectra of whole blood during open-heart surgery of goat. The *g* value^*^ and hyperfine splitting constant (hfsc)^¶^ indicate that the observed signals correspond to the ascorbyl free radical. Signals on both ends of each spectrum are those of the external standard of Mn^2+^. CPB: cardiopulmonary bypass.

Representative ESR spectra of goat blood during an open-heart surgery are shown in Figure 2.

**Figure 3:**
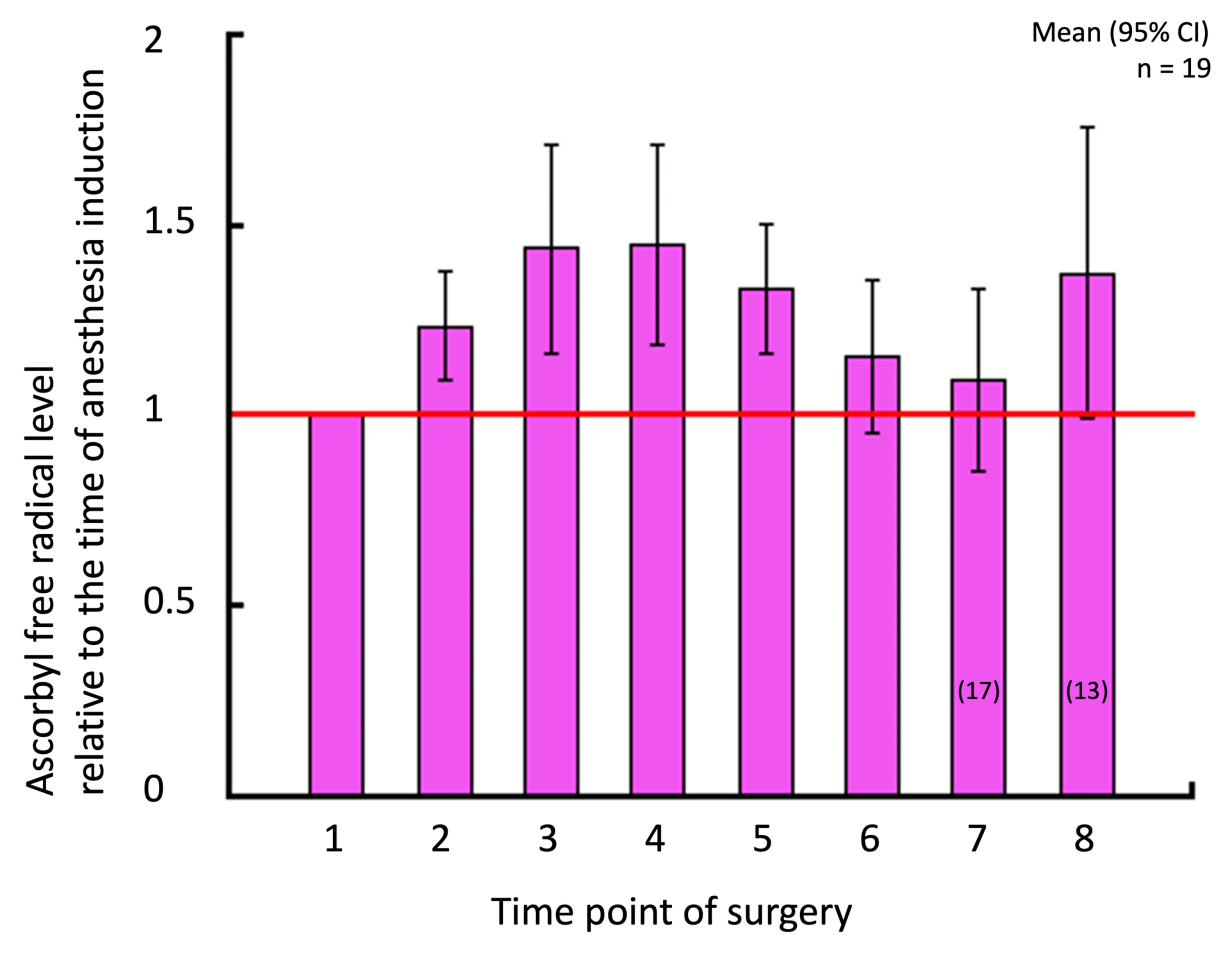
AFR levels detected in whole blood during open-heart surgery. The vertical axis indicates the ratios of the ESR signal heights (a.u.). Error bars indicate 95% confidence intervals (n = 19; n = 17 and 13 at the time points 7 and 8, respectively). The numbers along the horizontal axis correspond to those shown in Figure 2. AFR: ascorbyl free radical; ESR: electron spin resonance.

The ESR signals of AFR were consistently observed during the perioperative period. Figure 3 shows the average AFR levels at each time point during the open-heart surgery. Compared with the preoperative level, the AFR level detected in goat blood significantly increased from the start of CPB (95% CI: 1.09-1.39 ) and remained significantly higher than the preoperative level during aortic clamping (1.15-1.73 at aortic cross-clamping, 1.17-1.74 one hour after clamping, and 1.16-1.52 two hours after clamping). The AFR returned to the preoperative levels immediately after aortic declamping.

Free radical scavenging activity of perioperative plasma

**Figure 4:**
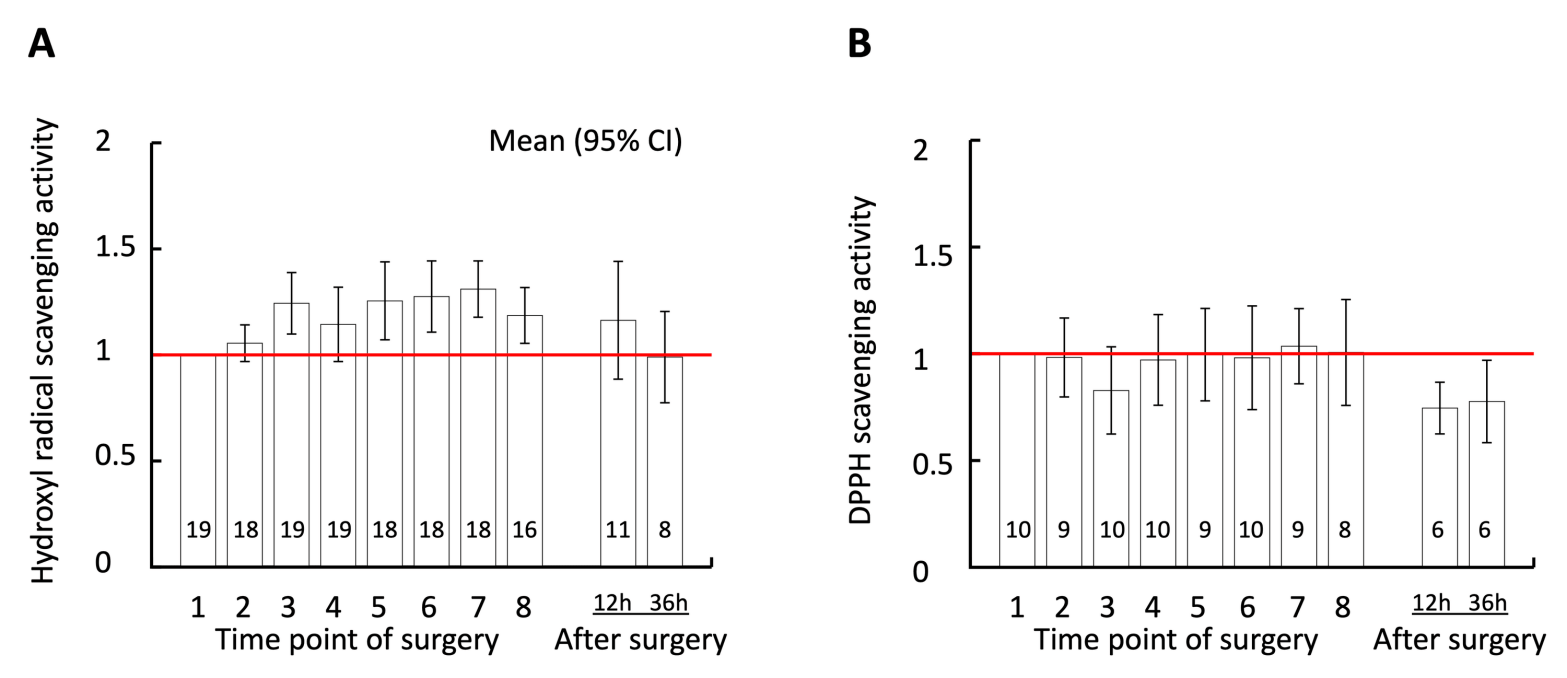
Free radical scavenging activity of plasma. The vertical axis shows radical scavenging activity of plasma relative to the time of induction of anesthesia against hydroxyl radicals (panel A) and 2,2-diphenyl-1-picrylhydrazyl (DPPH; panel B). The numbers on the horizontal axis correspond to those in Figures 2, 3. Error bars indicate 95% confidence intervals. The number in each column indicates the number of samples at each time point.

The free radical scavenging activities of plasma sampled at aortic cross-clamping (95% CI: 1.10-1.39) and from two hours after clamping (1.07-1.44) to the end of surgery (1.05-1.32) significantly increased against hydroxyl radicals (Figure 4). The scavenging activity returned to preoperative levels 12 hours after the end of surgery (0.89-1.44). In contrast, the scavenging activity against DPPH did not change throughout the surgery. However, 12 hours and 36 hours after the end of surgery, the scavenging activity significantly decreased to below the preoperative level (95% CI: 0.62-0.87 and 0.58-0.97, respectively).

## Discussion

The present study illustrated that the AFR level in goat whole blood significantly increased during the CPB procedure and returned to pre-CPB levels at aortic de-clamping (the end of aortic cross-clamping) and remained there until the end of surgery (Figure 3). Additionally, this study demonstrated that the free radical scavenging activity of perioperative plasma significantly increased against hydroxyl radicals but not against DPPH.

Excessive oxidative stress due to open-heart surgery leads to postoperative atrial fibrillation [11], postoperative delirium [12], and postoperative acute renal injury [13], impeding the prognosis. Establishing an antioxidative strategy to control oxidative stress during surgeries is the most pressing issue in anesthesiology, but it is not always successful [14]. One of the reasons for this failure might be that many surgical patients have comorbidities such as atherosclerosis, renal diseases, and diabetes mellitus, which correlate with oxidative stress at various severities [2]. In addition, operative time, anesthetic procedures, CPB, and aortic clamping may differ in each case. Real-time monitoring of oxidative stress during surgery might be essential for understanding the time course of oxidative stress during open-heart surgeries, which prompted us to conduct the present study. Real-time monitoring and quantification of oxidative stress by routinely used biomarkers such as malondialdehyde (MDA), 8-isoprostane, or total antioxidant capacity would be impossible. Since our AFR monitoring using whole blood can be measured in real-time with minimal sample preparation, it might be a very effective method for perioperative management in heart surgery under CPB.

Free radicals and reactive oxygen species produced in phagocytes are essential in immune responses. Free radicals are also essential as physiological signal transmitters *in vivo*. For example, the transcription factor nuclear factor erythroid 2-related factor 2 (Nrf2) plays a pivotal role in controlling the expression of antioxidant genes that ultimately exert anti-inflammatory functions [15]. However, excessive free radicals produced in the perioperative period injure live tissues and cells. For a successful antioxidative strategy in open-heart surgeries, it is crucial to maintain the balance between oxidative and reductive reactions *in vivo*.

Unlike the method described by Matsumoto et al. [9], where AFR levels were detected by adding dimethyl sulfoxide, the AFR signals detected in the present study were regarded as direct observations of the most downstream products produced in oxidative chain reactions *in vivo*. Thus, the observed increase in AFR in whole blood samples reflects an increase in the net production of free radicals in the whole body, including possibilities of localized myocardial or pulmonary radical generation influencing systemic levels. The increased production of free radicals may be related to perioperative stress. Thus, AFR detected in perioperative whole blood samples could be a sensitive real-time indicator of oxidative stress, which can be measured with a minimum preparation process in clinical settings.

It was reported that free radical scavenging capacity could change due to diseases, although the mechanism of such changes was unknown [16]. It was also suggested that oxidative stress and inflammatory processes might activate antioxidant processes, including gene expressions of Nrf2 [15]. This study illustrated the enhanced free-radical scavenging activity of plasma against hydroxyl radicals, which are the most harmful free radicals produced in the body. However, scavenging activity against DPPH, an artificial nitrogen-centered free radical, did not change, indicating that a change in free radical scavenging activity and antioxidant activity would not be induced uniformly. Although the metabolites responsible for such enhanced scavenging activity have not been elucidated yet, one possibility would be the increased production of ascorbate in the body of goats. Since this pathway does not exist in humans, translational extrapolation might require plasma supplementation models.

Direct detection of free radical species following reperfusion of the ischemic myocardium has been reported using the freeze-clamp ESR technique [17]. However, this technique requires sample processing, including pulverization of tissue prior to spectroscopy, which can artificially generate free radical species [17]. Application of the spin-trapping technique is another way of detecting free radicals. Arroyo et al. reported the successful detection of superoxide anions and hydroxyl radicals through spin trapping with DMPO (5,5-dimethyl-1-pyrroline N-oxide) [18]. Similarly, spin trapping using *N*-tert-butyl-α-phenylnitrone [19-21], DMPO [22-24], and 3,3,5,5-tetramethyl-1-pyrroline-*N*-oxid [25] have been used to detect free radicals *in vivo* animal models. However, our study aimed to observe oxidative stress in clinical settings; no spin-trapping agents would be administered to patients undergoing surgery. First, we attempted to directly detect free radicals by adding DMPO or CYPMPO to freshly sampled goat blood, which was unsuccessful. During those preliminary experiments, we observed AFR signals in fresh blood samples without adding DMPO [9], which prompted us to conduct the present study.

**Figure 5:**
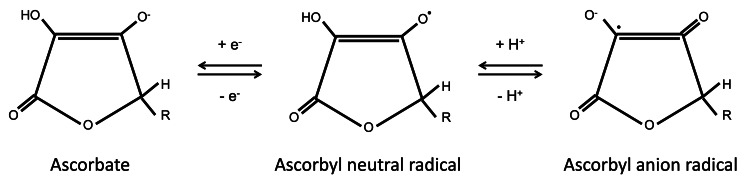
Chemical structures of ascorbate and ascorbyl free radicals.

The free-radical scavenging activity of ascorbate results from its non-enzymatic reduction of superoxide anions, hydroxyl, alkoxyl, peroxyl, and other free radicals [7]. Ascorbate also reduces tocopheroxyl radicals formed when tocopherol (vitamin E) reduces free radicals in lipid environments. These radicals take a single hydrogen atom from ascorbate and oxidize it to AFR (monodehydroascorbate; Figure 5). AFR, in turn, reacts preferentially with other radicals, forming dehydroascorbate in the process; AFR is not just a free radical scavenger but also a terminator of free radical chain reactions [26-29]. We initially speculated that the half-life of AFR was too short to be detected using ESR [7]; however, contrary to our assumption, AFR was detected in the whole blood of goats undergoing open-heart surgery. This finding indicates the possibility of real-time quantitative monitoring of the net amount of free radicals produced during a surgery by measuring AFR levels in whole blood.

In this study, we used an animal model of aortic valve replacement under CPB. In this model, heart ischemia began at the time of cross-clamping of the aorta after the initiation of CPB. Our results showed that the peak AFR level in the blood was observed before reperfusion, that is, one or two hours after cross-clamping. Interestingly, another peak was observed at the end of the surgery, i.e., not immediately after reperfusion. To the best of our knowledge, this is the first report of such a biphasic change in free radicals during heart surgery. It is speculated that the observed biphasic change may reflect increased production of free radicals not only due to ischemia/reperfusion injury of the myocardium but also due to systemic inflammatory reactions at CPB induction (e.g., hydroxyl radicals from neutrophils). At declamping of the aorta, the AFR level returned to the pre-CPB level, when the direct scavenging activity of the plasma against hydroxyl radicals was the highest. Another hypothesis would be the induction of antioxidative enzymes by Nrf2 attached to the antioxidant response elements due to excessive oxidative stress [15].

Hypothermia and the high partial pressure of oxygen during CPB can influence the redox state of animals. Hypothermia has been reported to be protective against oxidative stress. One study demonstrated that hypothermic conditions reduced oxidative stress during global cerebral ischemia/reperfusion injury, although it did not change the amount of reactive oxygen species significantly as assessed by ESR [30]. Meanwhile, hyperoxia induced increased production of superoxide anion, leading to an increase in downstream generation of reactive oxygen species and free radicals such as hydrogen peroxide and hydroxyl radical [31]. Thus, it is possible that some part of the increase in oxidative stress during CPB observed in the present study could be caused by CPB and hyperoxia, while hypothermal conditions were protective.

Based on the results of the present study, we propose the following antioxidative strategy for open-heart surgery under CPB. First, a dose of an antioxidant with already established safety (e.g., edaravone [10]) should be administered before CPB is started. Second, another dose of an antioxidant should be administered at the end of surgery. This strategy might enable efficient management of perioperative oxidative stress, thereby leading to improved prognosis. Unquestionably, types and doses of antioxidants should be investigated extensively before the clinical application of the strategy above. The present authors recommend the real-time monitoring of AFR presented here as a potential tool for such investigations.

The present study had several limitations. First, blood dilution by the crystalloid solution was necessary to prime the CPB circuit. However, AFR level and anti-hydroxyl radical scavenging activity significantly increased despite the dilution of blood, indicating an actual increase in AFR concentration and anti-hydroxyl radical scavenging activity *in vivo*. Second, the observed increase in the production of free radicals should be considered to be related to the entire surgical and anesthesiological procedures throughout the open-heart surgery and not just to ischemia/reperfusion injury of the heart. Third, it is impossible to specify which free radical species are present upstream of the oxidative chain reactions. Fourth, since goats are capable of synthesizing ascorbate *in vivo*, it is possible that ascorbate production increases in response to perioperative oxidative stress. However, it is unclear whether such an increase in production is possible within a few hours during the surgery. It would be recommended to directly measure plasma ascorbate concentrations in future experiments to confirm such compensatory synthesis. Finally, this study only evaluated the non-enzymatic free radical-scavenging activity of plasma. Potential enzymatic reactions in the plasma were not considered.

## Conclusions

We conducted open-heart surgeries in goats and successfully observed an increase in AFR in fresh whole blood, which reflects an increase in the production of free radicals under perioperative oxidative stress. This simple measurement, without a spin trap or an enhancer, can be implemented at a low cost. This may enable novel real-time monitoring of oxidative stress in perioperative patients, although feasibility testing in human blood *ex vivo* is required as the next step. Such an application could significantly contribute to the control of oxidative stress and thus prevent complications.
